# Excitability is increased in hippocampal CA1 pyramidal cells of *Fmr1* knockout mice

**DOI:** 10.1371/journal.pone.0185067

**Published:** 2017-09-20

**Authors:** M. Angeles Luque, Pablo Beltran-Matas, M. Carmen Marin, Blas Torres, Luis Herrero

**Affiliations:** Department of Physiology. University of Seville, Seville, Spain; University Paris 6, FRANCE

## Abstract

Fragile X syndrome (FXS) is caused by a failure of neuronal cells to express the gene encoding the fragile mental retardation protein (FMRP). Clinical features of the syndrome include intellectual disability, learning impairment, hyperactivity, seizures and anxiety. *Fmr1* knockout (KO) mice do not express FMRP and, as a result, reproduce some FXS behavioral abnormalities. While intrinsic and synaptic properties of excitatory cells in various part of the brain have been studied in *Fmr1* KO mice, a thorough analysis of action potential characteristics and input-output function of CA1 pyramidal cells in this model is lacking. With a view to determining the effects of the absence of FMRP on cell excitability, we studied rheobase, action potential duration, firing frequency–current intensity relationship and action potential after-hyperpolarization (AHP) in CA1 pyramidal cells of the hippocampus of wild type (WT) and *Fmr1* KO male mice. Brain slices were prepared from 8- to 12-week-old mice and the electrophysiological properties of cells recorded. Cells from both groups had similar resting membrane potentials. In the absence of FMRP expression, cells had a significantly higher input resistance, while voltage threshold and depolarization voltage were similar in WT and *Fmr1* KO cell groups. No changes were observed in rheobase. The action potential duration was longer in the *Fmr1* KO cell group, and the action potential firing frequency evoked by current steps of the same intensity was higher. Moreover, the gain (slope) of the relationship between firing frequency and injected current was 1.25-fold higher in the *Fmr1* KO cell group. Finally, AHP amplitude was significantly reduced in the *Fmr1* KO cell group. According to these data, FMRP absence increases excitability in hippocampal CA1 pyramidal cells.

## Introduction

Fragile X syndrome (FXS) is the most common form of inherited human intellectual disability. Many FXS patients display learning impairment, hyperactivity, hypersensitivity to sensory stimuli, seizures and anxiety. Thirty percent of children with FXS are diagnosed with autism [1]. FXS is caused by transcriptional silencing of the *FMR1* gene which encodes the fragile mental retardation protein (FMRP). *Fmr1* knockout (KO) mice do not express FMRP, and reproduce some of the behavioral abnormalities seen in FXS; these animals are commonly used as a model to understand the molecular-, synaptic-, cellular-, and neural network-bases of the syndrome [2–7].

Electrophysiological research carried out on brain tissue from *Fmr1* KO mice has identified impairment of long- and short-term synaptic plasticity [8–10], abnormal dendritic excitability associated with alterations in the expression and/or function of several types of voltage-gated ion channels [11–15], and presynaptic dysfunction dependent on N-type voltage-gated calcium channels [16]. The abnormal dendritic excitability attributed to ion channels appears to be specific both to the brain region and to the cell type under investigation [13, 15, 17]. Studies of intrinsic excitability using somatic patch-clamp recordings have also been carried out. Some reports suggested unaltered membrane properties of layer 5 pyramidal neurons in the somatosensory cortex [15, 18]. On the other hand, an increased input resistance probably underlies a decrease of the minimum current step required to evoke an action potential and the increased firing frequency seen in response to a given suprathreshold current injection in layer 4 excitatory neurons in the barrel sensory cortex [19]. This neuronal population, under an epileptiform condition, switch from a regular spiking pattern to a seizure-like activity [20]. An absence of FMRP increased the persistent sodium current which diminished action potential threshold and caused pyramidal cell hyperexcitability in the entorhinal cortex [21]. Layer 2/3 neurons of the prefrontal cortex in *Fmr1* KO mice display a higher excitability as measured at the soma, which could result from a larger transient Na^+^ current and a depolarizing shift in the activation of A-type K^+^ conductance [22]. Action potential broadening, via a reduction in the activity of BK channels, has been reported in layer 5 pyramidal cells of the entorhinal cortex and in CA3 pyramidal neurons of the hippocampus in *Fmr1* KO mice [23]. In this way, a primary objective of the current work was to increase our present understanding of cell excitability by studying the hippocampal CA1 pyramidal neurons in wild-type (WT) and *Fmr1* KO mice.

The hippocampus is widely recognized as a critical structure for learning and memory; cell hyperexcitability could, at least in part, underlie behavioral deficits associated with the absence of FMRP [for review see 5, 6, 7]. FMRP is highly expressed in the somatodendritic domains of neurons in all hippocampal areas [24] and acts through multiple mechanisms, including as a translational regulator of its mRNA targets, some of which, encode voltage-gated ion channels, and interaction with ion channel-associated proteins [11, 16, 23, 25–29]. In physiological conditions, cell bodies integrate incoming signals from synaptic transmission. Abnormal channel expression and/or function can have substantial effects on the processes of cell body signal integration and the generation of neuronal outputs [30]. The manner in which the absence of FMRP can affect cell computations can be captured by the input–output function. The input–output function is characterized by the threshold and the gain [31]. Here we define the threshold from rheobase and the gain from the slope of the relationship between firing frequency and injected current. Modifications in resting membrane potential, input resistance, and /or action potential voltage threshold could shift rheobase, and therefore increase or decrease cell excitability. A change in the slope (gain) can lead to the amplification or scaling-down of the sensitivity of the neuron to stimuli [31, 32]. On the other hand, FMRP-dependent alterations in action potential duration can modify neurotransmitter release [23]. Based on this knowledge, we investigated whether the absence of FMRP by genetic modification alters rheobase, the firing frequency–current injection relationship, and/or the shape of the action potential, including after-hyperpolarization (AHP).

## Materials and methods

### Slice preparation

*Fmr1* KO mice (FVB.129P2-*Pde6b*^*+*^ Tyr^c-ch^ *Fmr1*^*tm1Cgr*^/J; stock# 4624, Jackson Laboratory) and wild-type (WT) mice (FVB.129P2-*Pde6b*^*+*^*Tyr*^*c-ch*^ /AntJ; stock# 4828, Jackson Laboratory) were used in these studies. All experimental procedures were approved by the Ethics Committee on Animal Experimentation at the University of Seville (Permit number 12-06-15.260) in accordance with the guidelines of the European Community (Directive 2010/63/EU), as well as with the Spanish legislation (R.D. 53/2013 BOE 34/11370-421). Male mice (8–12 weeks old) were anesthetized deeply with sodium pentobarbital (50 mg kg-1) and all efforts were made to minimize suffering. Mice were then perfused intracardially with ice-cold sucrose-artificial cerebrospinal fluid (ACSF) and decapitated; brains were removed quickly and placed in a dissection medium containing cold sucrose–ACSF. Coronal slices (300-μm thickness) including the hippocampi were cut using a vibrating microtome (Leica 1200S). Slices were first incubated in a chamber containing ACSF for 30–45 minutes at 32°C, and then maintained at room temperature. Single slices were transferred to the recording chamber and superfused at 2 ml min^-1^ with ACSF bubbled with 95% O2–5% CO2 (pH 7.4; 32 ± 1°C). The composition of ACSF was as follows (in mM): 126 NaCl, 2 KCl, 1.25 Na2HPO4, 26 NaHCO3, 10 glucose, 2 MgSO4, and 2 CaCl_2_. For the sucrose-ACSF solution, NaCl was substituted by sucrose (240 mM) and the concentrations of MgSO_4_ and CaCl_2_ were modified to 4 mM and 0.1 mM, respectively.

### Electrophysiological recordings

Whole-cell patch clamping was performed on visually identified somata of CA1 pyramidal cells using a Nikon Eclipse FN1 equipped with infrared-differential interference contrast optics, a 40× water immersion objective and a Hamamatsu C-7500 camera. Recordings were carried out on neurons located approximately between Bregma -1.3 and -1.9 mm [33] in the dorsal region of the hippocampus. For current clamp recordings, patch pipettes (4–6 MΩ) contained (in mM): 120 potassium gluconate, 10 KCl, 10 phosphocreatine disodium salt, 2 MgATP, 0.3 NaGTP, 0.1 EGTA and 10 Hepes, adjusted to pH 7.3 with KOH. The osmolality of intracellular solutions was 280–290 mosmol l^−1^, adjusted with sucrose. Whole-cell patch clamp recordings were carried out using a MultiClamp 700B Amplifier (Molecular Devices). Gigaseals (>1 GΩ) were obtained before rupture of the patch, pipette capacitance was compensated before breaking in and the bridge was balanced using the auto-adjust button. Series resistance was 20 MΩ or less during recording. Current clamp recordings were low-pass Bessel-filtered at 10 kHz; data were digitized at 20 kHz with a Digidata 1440A analog-to-digital converter and acquired using pCLAMP 10 software (Molecular Devices). Data were stored on a computer disk and analyzed offline using Clampfit 10.2 (Molecular Devices) software. Voltages were not corrected for the liquid-junction potential (estimated as ~14 mV).

### Data acquisition and analysis

Current clamp experiments were conducted to determine whether FMRP modulates the cell excitability of hippocampal CA1 pyramidal cells and a number of parameters were quantified. Resting membrane potentials were measured as the difference between the intracellular and extracellular potentials. Input resistance was determined by passing current steps (500 ms, 1 Hz; with 10 pA increments from 20 to—60 pA), and calculated as the slope of the current-voltage plot. To measure inward rectification, or *sag*, a current pulse that drifted the membrane potential from rest to -85 ± 2 mV was applied; *sag* amplitude was calculated as the difference between membrane potentials at peak and at steady-state; the percentage of sag was equal to (sag amplitude / voltage difference between rest and peak amplitude) x 100. The membrane time constant was obtained by recording the membrane response to 20 pA hyperpolarizing current pulses (500 ms duration, 1 Hz) and fitting the response to a single exponential curve. We chose 20 pA of hyperpolarizing current because such a current intensity did not produce sag. The rheobase was the minimum injected current that generated an action potential in 50% of cases, and was measured for pulse durations of 500 ms (1Hz; with 2 pA increments). Depolarization voltage was the increase in membrane potential required for the cell to reach the spike voltage threshold. To assess the spike voltage threshold, we used phase-plane plots and the spike onset was taken as the value of the membrane potential at which the first derivative exceeded 10 V s^−1^. Single action potentials were evoked by short (100 μs) depolarizing current pulses; the current intensity was progressively increased by 0.5 nA current steps until the spike voltage threshold was reached. Action potential amplitude was the voltage increment between the resting level and spike voltage peak. The value of the duration of the action potential was determined as the width of the spike at half-maximal amplitude. Repetitive firing was evoked by depolarizing current steps (500 ms, 0.5 Hz) with 10–50 pA increments, and the mean firing for each injected current in WT and *Fmr1* KO cells was calculated. Next, the relationship between the firing frequency and injected current was plotted for each cell and the slope (gain) determined. Evoked action potentials were followed by an after-depolarization (ADP) and /or AHP. A 100 μs stimulus with current intensity adjusted just above the spike voltage threshold yielded an action potential often followed by an ADP, the amplitude of which was taken as the voltage difference between rest and membrane potential peak during ADP. The duration of the ADP corresponded to the time between ADP onset and rest recovery. The amplitude and /or duration of the fast and medium components of the action potential AHP were also measured [34]. AHP parameters were quantified for three conditions: (1) with current depolarizing pulses of 500 ms that shifted the membrane potential to the spike threshold value and evoked spikes at a low firing frequency (≤ 2 action potential s^-1^); (2) with suprathreshold current steps lasting 500 ms and producing 8–12 action potentials; and (3) at the end of the repetitive discharge evoked by the previous current injections. For low firing frequency, fast AHP amplitude was the difference between resting membrane potential and the lowest value of membrane potential during this AHP phase. The amplitude of the medium AHP was measured as the difference between rest and membrane potential in a time window of 5–15 ms after the lowest value of membrane potential during fast AHP. To measure the medium AHP component after repetitive discharge, the amplitude and duration were assessed relative to the resting membrane potential. For spike trains, AHP amplitudes were assessed relative to the action potential threshold. To study spike-frequency adaptation, the instantaneous frequency was calculated as the reciprocal of the inter-spike interval duration. The spike-frequency adaptation was then quantified by the adaptation index, which is equal to [1—(frequency in the steady-state ⁄ instantaneous frequency in the first inter-spike interval)]. The steady-state firing frequency was taken as the average of the instantaneous frequency during the last 250 ms of the repetitive discharge. Adaptation index was measured from trains of 8–12 action potentials evoked by 500 ms current injections.

### Statistical analysis

Significant differences between WT and *Fmr1* KO cell groups were determined by using the Student’s t test for unpaired samples as values were normally distributed within each group with equal variance between groups. The correlation between variables was measured by Pearson’s correlation coefficient (r). The significance level was established at P < 0.05. All data are reported as mean ± standard error of the mean. The percentage change in each parameter was normalized relative to the WT cell group and calculated as: (*Fmr1* KO mean value—WT mean value / WT mean value) x 100.

## Results

### Rheobase in CA1 pyramidal cells is not modified in *Fmr1* KO mice

**Fig 1:**
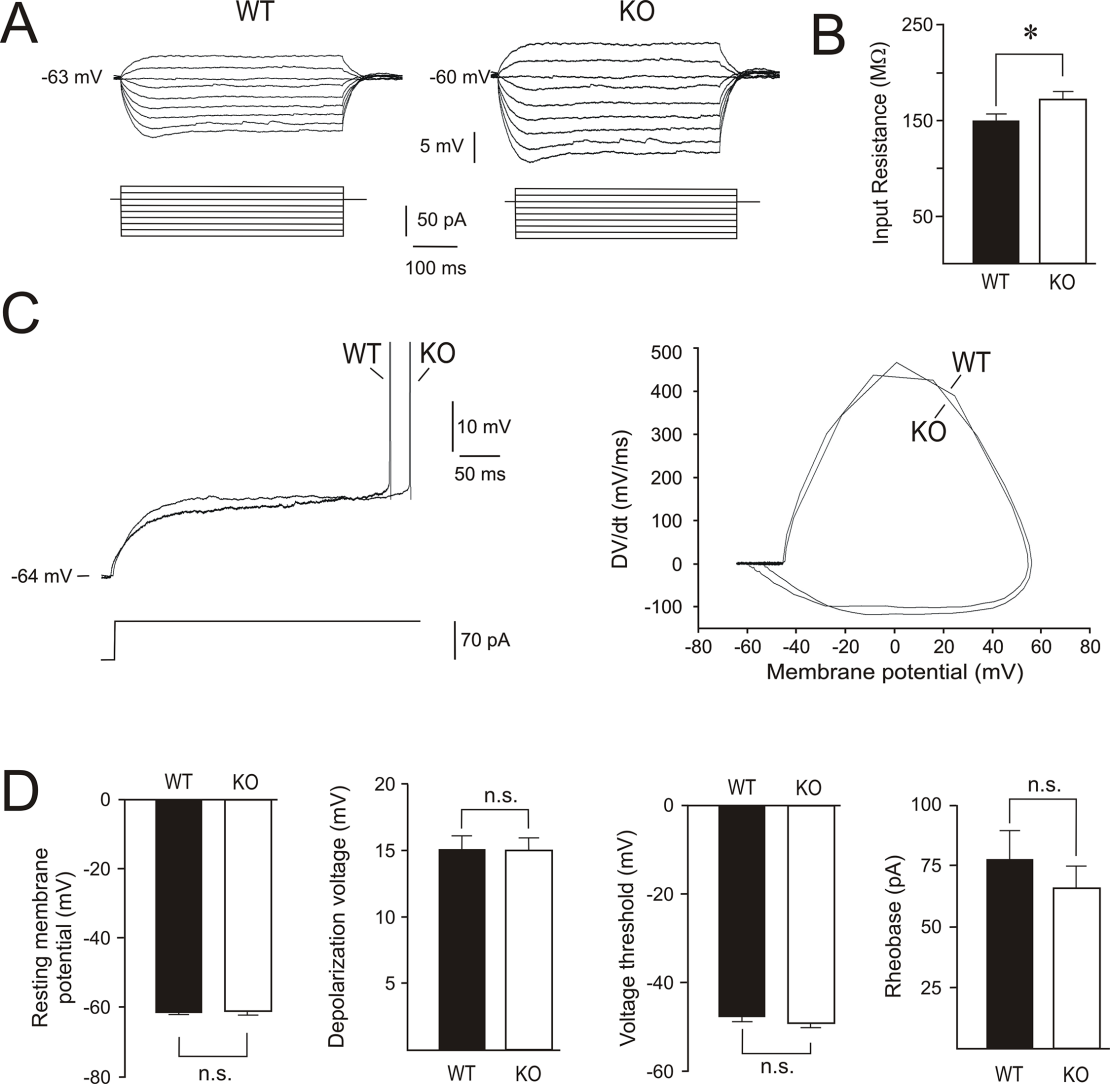
Rheobase is not modified in CA1 pyramidal cells of *Fmr1* KO mice. (A) Voltage responses of two representative WT and *Fmr1* KO cells to depolarizing and hyperpolarizing current steps. (B) Histogram showing input resistance in WT and *Fmr1* KO cell groups. (C) Representative recordings of the rheobase that was 70 pA for both WT and *Fmr1* KO cells (left panel). Phase-plane plots to determine the action potential voltage threshold (right panel). (D) Histograms showing resting membrane potential, depolarization voltage, action potential voltage threshold and rheobase in WT and *Fmr1* KO cell groups. Calibrations as indicated. In this and following figures, histograms show the mean value and standard error of the mean. Asterisks (*) indicate statistically significant differences (P<0.05). n.s. indicates non-significant statistical differences.

Whole-cell patch clamp recordings were performed on the somas of 33 WT and 32 *Fmr1* KO pyramidal cells located within the hippocampal CA1 region. These cells were from slices prepared from 14 WT and 16 *Fmr1* KO mice (details in S1 and S2 Tables). Resting membrane potential, input resistance and action potential voltage threshold were measured to assess if the absence of FMRP leads to changes in rheobase. All cells included for analysis showed a stable resting membrane potential, and the mean value of the resting membrane potential was close to—63 mV in both cell groups (Fig 1D; Table 1). The *Fmr1* KO cell group exhibited a significantly higher input resistance (~15%; Fig 1A and 1B; Table 1). The amplitude of *sag* was measured, as the altered functional expression of h-channels in dendrites has been linked to input resistance modifications in *Fmr1* KO mice [13, 15], and there were no differences between WT and *Fmr1* KO cell groups (Table 1). The *Fmr1* KO cell group showed a significantly higher membrane time constant (~15%) than the WT cell group (Table 1). Next, we measured whether spike voltage threshold and depolarization voltage (i.e. the difference between spike voltage threshold and resting membrane potential) were different between WT and *Fmr1* KO cell groups and found non-significant differences (Fig 1C and 1D; Table 1). Higher values of input resistance in the *Fmr1* KO cell group were accompanied by a reduction of rheobase (in the *Fmr1* KO group was ~13% lower than in the WT group); however, differences in rheobase were not statistically significant (Fig 1D; Table 1).

**Table 1:** Membrane properties of the CA1 pyramidal cells in wild type and *Fmr1* KO mice.

Parameter	Wild type mice (n)^1^	*Fmr1* KO mice (n)^1^	Statistics^2^ (P)*	% Change^3^
Resting membrane potential (mV)	-63.33 ± 0.49 (33/14)	-63.02 ± 0.87 (32/16)	0.760	-0.50
Input resistance (MΩ)	151.8 ± 6.90 (32/13)	174.1 ± 8.16 (32/16)	0.040*	14.69
Time constant (ms)	20.41 ± 0.78 (32/13)	23.39 ± 0.93 (32/16)	0.017*	14.60
Sag (%)	16.42 ±0.72 (13/9)	13.91 ± 0.75 (21/11)	0.030	-9.19
Rheobase (pA)	76.64 ± 11.68 (22/10)	66.94 ± 9.02 (17/11)	0.538	-12.65
Voltage threshold (mV)	-47.62 ± 1.06 (22/10)	-49.03 ± 0.89 (17/11)	0.336	2.96
Depolarization voltage (mV)	15.24 ± 1.29 (22/10)	15.15 ± 1.23 (17/11)	0.963	-0.59
Action potential duration^4^ (ms)				
Single action potential	0.99 ± 0.01 (28/11)	1.06 ± 0.02 (29/14)	0.026*	7.07
Firing train (8–12 AP during 500 ms)				
First	0.96 ± 0.02 (12/9)	1.02 ± 0.01 (11/8)	0.046*	6.25
Middle	0.98 ± 0.01 (12/9)	1.04 ± 0.01 (11/8)	0.035*	6.12
Last	0.99 ± 0.01 (12/9)	1.03 ± 0.01 (11/8)	0.041*	4.04
Firing frequency^5^ (AP • s^-1^)				
100 pA	10.64 ± 1.71 (22/11)	15.58 ± 1.58 (19/13)	0.042*	46.42
200 pA	22.55 ± 2.04 (22/11)	31.37 ± 2.21 (19/13)	0.005*	39.11
300 pA	32.57 ± 2.16 (22/11)	41.05 ± 1.79 (19/12)	0.006*	26.03
400 pA	38.80 ± 2.44 (20/10)	45.05 ± 1.75 (19/12)	0.046*	16.10
Gain^6^	0.12 ± 0.006 (22/11)	0.15 ± 0.006 (19/12)	0.013*	25
ADP/AHP^7^				
ADP single action potential				
Amplitude (mV)	8.01 ± 0.66 (24/14)	6.93 ± 0.55 (26/13	0.220	-13.48
Duration (ms)	65.96 ± 4.08 (24/14)	92.89 ± 7.49 (26/13)	0.003*	40.82
AHP low frequency (≤2 AP • s^-1^)				
fAHP Amplitude (mV)	10.29 ± 0.92 (22/11)	7.68 ± 0.80 (17/11)	0.047*	-25.36
fAHP Duration (ms)	3.33 ± 0.41 (22/11)	3.52 ± 0.19 (17/11)	0.709	5.70
mAHP Amplitude (mV)	7.57 ± 0.76 (22/11)	4.40 ± 0.57 (16/11)	0.003*	-41.87
mAHP Duration (ms)	167.73 ± 9.65 (22/11)	153.5 ± 16.63 (15/11)	0.436	-8.84
AHP firing train (8–12 AP during 500 ms)				
fAHP Amplitude (mV)	10.67 ± 1.214 (12/9)	7.72 ± 0.59 (11/8)	0.044*	-27.64
fAHP Duration (ms)	2.05 ± 0.19 (12/9)	3.17 ± 0.12 (11/8)	0.001*	54.63
mAHP Amplitude (mV)	9.64 ± 1.32 (12/9)	6.13 ± 0.90 (11/8)	0.043*	-36.41
mAHP Duration (ms)	58.28 ± 2.9 (12/9)	50.39 ± 3.64 (11/8)	0.102	-13.53
mAHP after a firing train (8–12 AP during 500 ms)				
Amplitude (mV)	2.15 ± 0.16 (5/3)	1.39 ± 0.24 (4/3)	0.029*	-35.34
Duration (ms)	121.2 ± 16.29 (5/3)	141.78 ± 25.3 (4/3)	0.498	16.98
Adaptation index^8^	0.46 ± 0.06 (12/9)	0. 43 ± 0.06 (11/8)	0.763	-6.52

^1 ^(n) indicates the number of cells / mice quantified for each parameter.

^2^ Statistical significance level (P value) after comparing mean values using Student’s t test for unpaired samples

* indicates significant differences between both cell groups (P< 0.05).

^3^ Data in this column correspond to the percentage of change between the mean values from *Fmr1* KO cells compared to WT cells and was calculated as: (*Fmr1* KO mean value—WT mean value / WT mean value) x 100. Positive and negative values indicate increments and decrements of *Fmr1* KO cell group relative to WT cell group.

^4^ Action potential (AP) durations were measured after short stimuli (100 μs–single AP) and during 500 ms depolarizing current pulses.

^5^ Action potential firing frequencies were evoked by 500 ms depolarizing current steps at 100, 200, 300 and 400 pA and measured as action potentials (AP) per second.

^6^ Gain was the slope of the linear relationship between the firing frequency and injected current.

^7^ Action potentials after-depolarization (ADP) and after-hyperpolarization (AHP) were measured. Fast (fAHP) and medium (mAHP) were quantified for two conditions: with current steps of 500 ms that shifted membrane potential to the spike voltage threshold and evoked spikes at a low firing frequency (< 2 action potential • s^-1^); with suprathreshold current steps that evoked 8–12 spikes. At the end of the repetitive discharge evoked by the previous suprathreshold current, mAHP was also quantified.

^8^ The adaptation index was calculated as [1—(frequency in the steady-state ⁄ instantaneous frequency in the first inter-spike interval)]. The repetitive discharge (8–12 AP) was evoked by 500 ms depolarizing current steps.

### Action potential duration is longer in CA1 pyramidal cells from *Fmr1* KO mice

**Fig 2:**
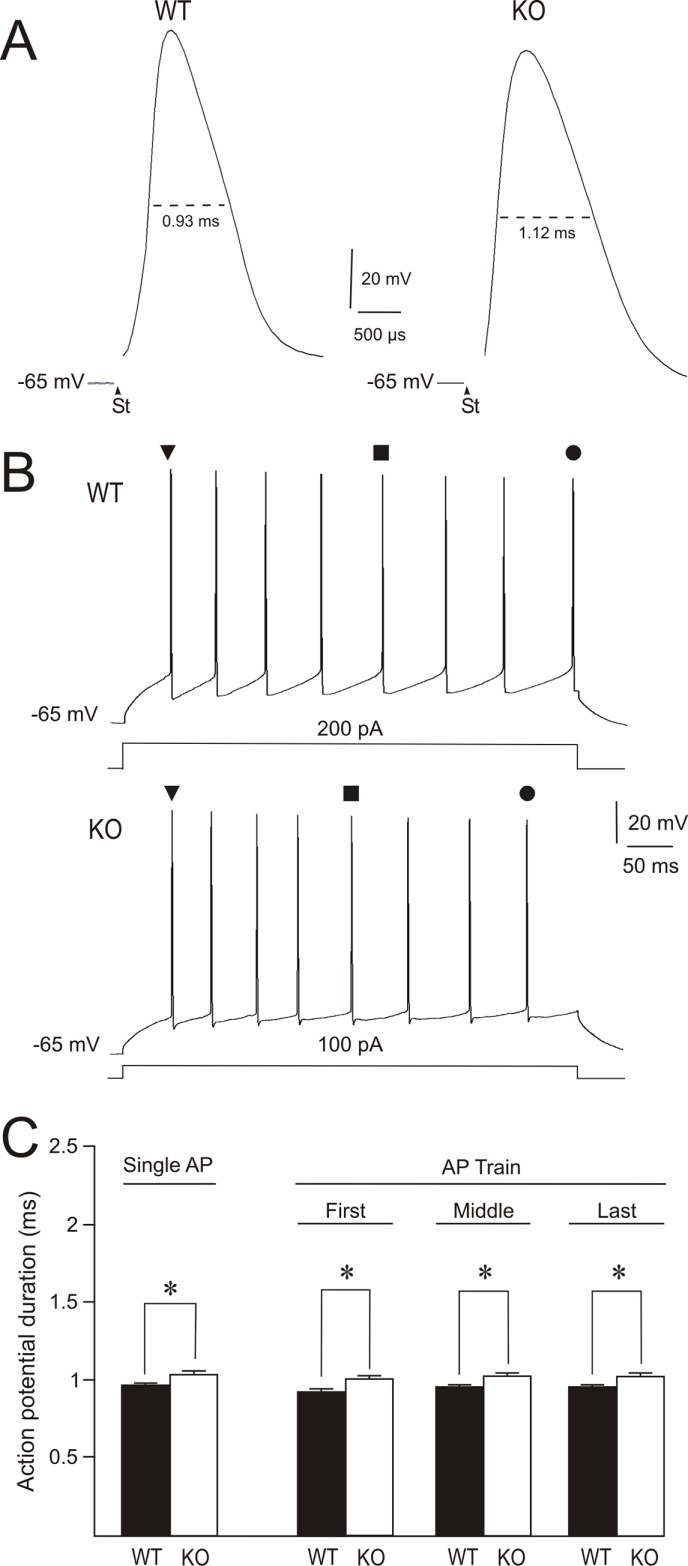
Action potential duration is increased in CA1 pyramidal cells of *Fmr1* KO mice. (A) Single action potential in a WT and a *Fmr1* KO cell generated by a short pulse (100 μs) with an intensity that was adjusted to the spike voltage threshold. The recordings also depict the half-width (dashed line) of the spike for the measurement of action potential duration. St indicates the onset of the stimulus. (B) Train of action potentials (8–12 spikes) produced by applying 500 ms current injections at 200 pA in a WT cell and at 100 pA in a *Fmr1* KO cell. The durations of the first spike (▼), the spike in the middle of the train (250 ms after stimulus onset; ■) and the last spike of the train (●) were measured in both cell groups. (C) Histogram showing action potential duration evoked in a single action potential and in trains of action potentials in WT and *Fmr1* KO cell groups. Calibrations as indicated. Asterisks (*) indicate statistically significant differences (P<0.05).

Action potential broadening associated with the absence of FMRP was recently reported for pyramidal cells within CA3 region of the hippocampus [23]. When this parameter was assessed in relation to excitatory neurons located in layer 5 of the entorhinal cortex, the magnitude of the effect of FMRP absence on the action potential duration appeared to fall into two different cell populations: one of these populations showed action potential broadening similar to CA3 pyramidal cells, whereas the other population exhibited highly exaggerated broadening. According to these reports, the absence of FMRP produces an increased action potential duration, the magnitude of which depends on the cell population under investigation. To determine if such a finding can be extended to CA1 pyramidal cells, the action potential duration was assessed following short- and long-duration current steps. Short current steps (100 μs) evoked single action potentials. These spikes were lower in amplitude (WT = 118.56 ± 1.51 mV from 28 cells of 11 mice versus *Fmr1* KO = 105.9 ± 2.22 from 29 cells of 14 mice) and longer in duration (~7%) in cells from the *Fmr1* KO group compared to cells from WT mice (Fig 2A; Table 1). Differences between groups were statistically significant for both parameters. Because action potential duration increases during burst firing [23], 500 ms current steps were injected to evoke trains of 8–12 action potentials and the durations of the first, middle (250 ms after stimulus onset) and last spike of the train were measured in both cell populations. Action potentials were longer in duration (~6%) in the *Fmr1* KO cell population compared with the WT cell group (Fig 2B and 2C; Table 1). In conclusion, the absence of FMRP results in an increased action potential duration.

### The slope (gain) of the firing frequency–current intensity relationship is higher in CA1 pyramidal cells from *Fmr1* KO mice

**Fig 3:**
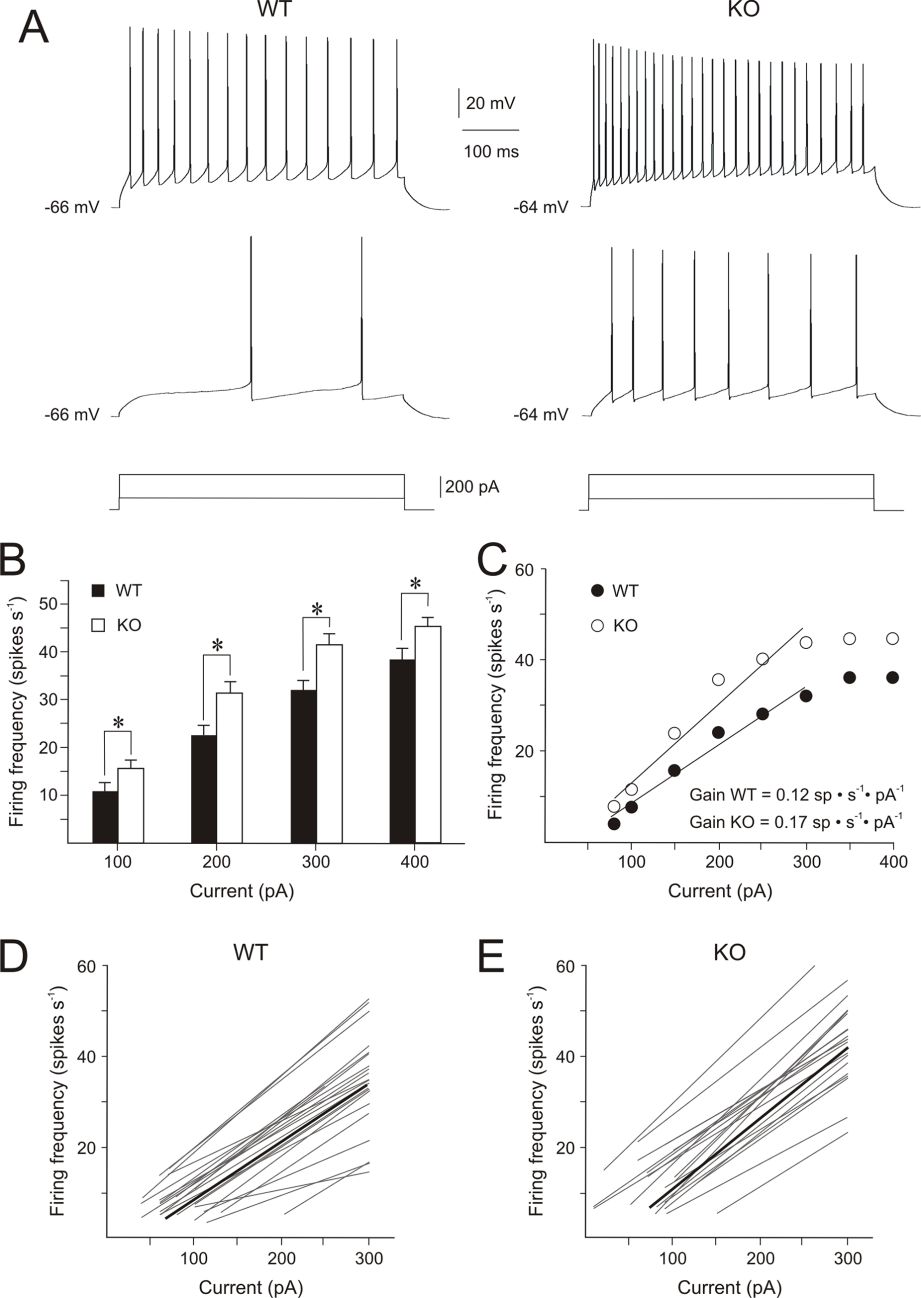
Action potential firing frequency is increased in CA1 pyramidal cells of *Fmr1* KO mice. (A) Firing frequency depended on injected current in both WT and *Fmr1* KO cells, but in response to the same current intensity the number of spikes was higher in *Fmr1* KO cells. (B) Histogram showing firing frequency (spikes s^-1^) evoked by current steps of 100, 200, 300 and 400 pA in WT and *Fmr1* KO cell groups. (C) Plot of the firing frequency versus injected current relationship for representative WT and *Fmr1* KO cells. The gain corresponds to the slope of the linear relationships. (D, E) Linear relationships between injected current and firing frequency for WT and *Fmr1* KO cells are represented in grey color. The black lines correspond to the mean fits for each group: WT, firing frequency = 0.12 ∙ injected current—2.7; *Fmr1* KO, firing frequency = 0.15∙ injected current—3.7. Calibrations as indicated. Asterisks (*) indicate statistically significant differences (P<0.05).

CA1 pyramidal cells showed a repetitive discharge of action potentials in response to suprathreshold depolarizing current steps. Current steps of equal intensity produced a higher number of spikes in *Fmr1* KO cells than in WT cells (Fig 3A and 3B). The increased firing frequency of *Fmr1* KO cells was statistically significant for current steps of 100, 200, 300 and 400 pA (Fig 3B; Table 1). Fig 3C shows the firing frequency–current intensity relationships of representative WT and *Fmr1* KO cells. As shown, firing frequency increases with the stimulus intensity, reaching a firing plateau with stimuli ≥ 300 pA. For this reason, data were fitted to a linear regression up to 300 pA. Both WT and *Fmr1* KO neurons had a good fit (r> 0,9), and *Fmr1* KO cell group exhibited a significantly higher slope (gain) than WT (Fig 3D and 3E; Table 1). The mean gain in *Fmr1* KO cell group was 1.25-fold higher than WT cell group. Next, we studied spike-frequency adaptation and found that these were similar (~ 0.45) in both cell groups (Table 1). In summary, the absence of FMRP resulted in an 1.25-fold increase in the gain of the firing frequency–current intensity relationship; this alteration leads to a heightened sensitivity of *Fmr1* KO cells to changes in input.

**Fig 4:**
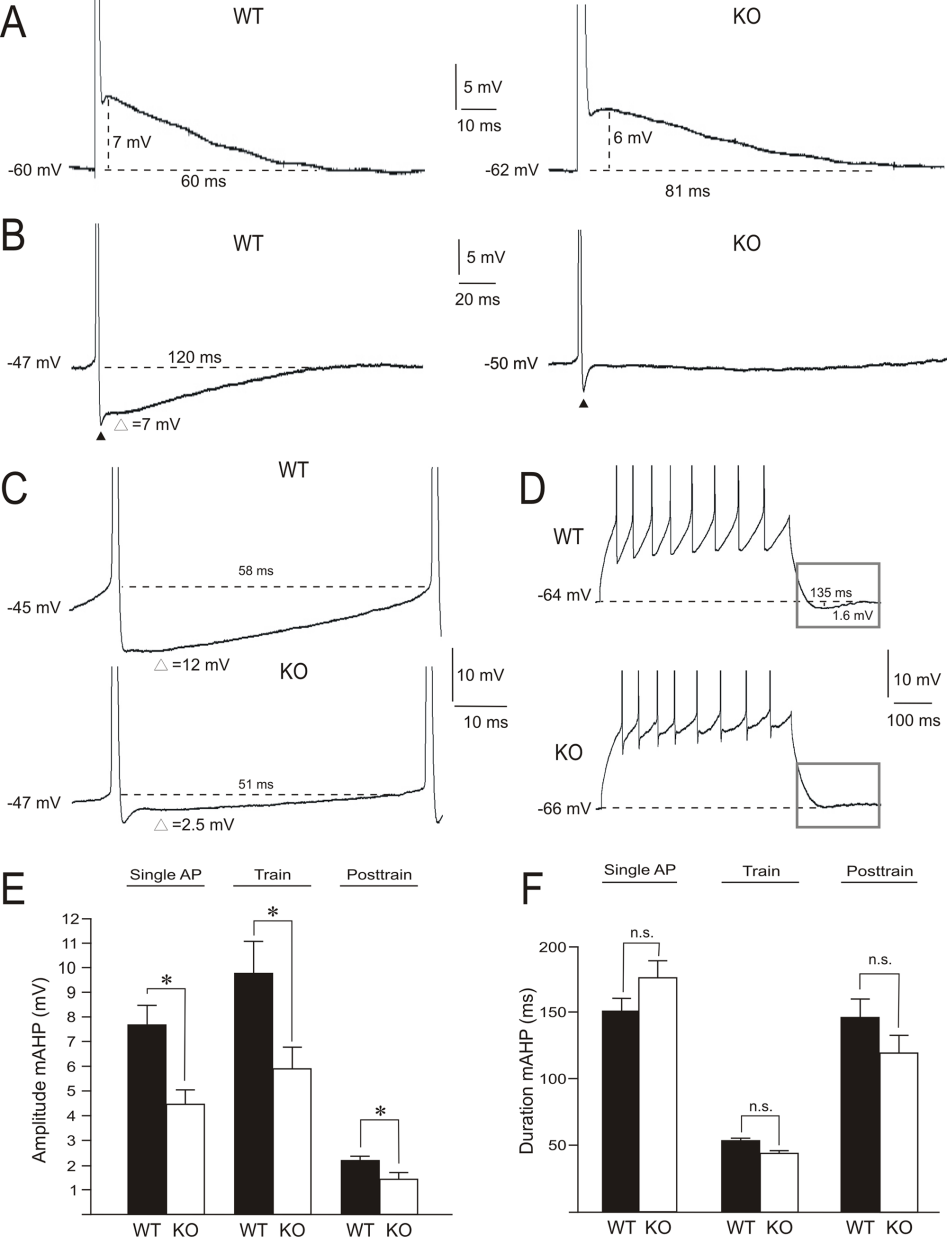
Amplitude of the action potential fast and medium after-hyperpolarization (AHP) diminished in CA1 pyramidal cells of *Fmr1* KO mice. (A) Action potential after-depolarization in response to a short stimulus (100 μs, current intensity adjusted to spike voltage threshold). (B) Action potential AHP in response to depolarizing current steps of 500 ms that shifted the membrane potential to the spike voltage threshold and produced action potentials at low firing frequencies (≤ 2 spikes s^-1^). It should be noted that fast (▲), but not medium (∆), AHPs were observable in *Fmr1* KO cells. (C) Amplitude and duration of the medium AHP during a repetitive firing, measured in the spike closest to 250 ms after stimulus onset. Current intensity was adjusted to evoke similar number of spikes (8–12 action potentials for 500 ms pulse durations) in both cell groups. (D) Medium AHP after a train of action potentials (area indicated by square) in WT and *Fmr1* KO cells. Note the absence of a medium AHP in the *Fmr1* KO cell. (E, F) Histograms show the amplitude and duration of medium AHPs in WT and *Fmr1* KO cell groups. Calibrations as indicated. Asterisks (*) indicate statistically significant differences (P<0.05). n.s. indicates non-significant statistical differences.

CA1 pyramidal cells showed an action potential ADP and AHP in response to short and long suprathreshold current steps, respectively (Fig 4). Stimuli of 100 μs duration evoked single action potentials that were often followed by an ADP (n = 24 of 28 WT cells and n = 26 of 29 F*mr1* KO cells). The amplitude of this response was similar in *Fmr1* KO and WT cells, but the ADP duration was significantly longer in *Fmr1* KO cells (Fig 4A; Table 1). The injection of 500 ms depolarizing current steps shifted the membrane potential to values close to the spike threshold, yielding action potentials at low firing frequencies (≤ 2 action potentials s^-1^). Under this condition, AHP recordings were noticeably different in cells from the two groups. WT cells exhibited a fast and a medium AHP, as already reported in rat CA1 pyramidal cells [33]. In *Fmr1* KO cells, fast AHPs were observed, whereas medium AHPs were absent or weak (Fig 4B). Consistent with previously reported data for CA3 pyramidal cells [23], an absence of FMRP causes a significant reduction of the amplitude of fast AHPs in CA1 pyramidal cells (Table 1). Fast and medium AHP characteristics were also studied during repetitive discharge. Because firing frequency produces different amounts of calcium influx which can shape AHP [35, 36], trains of 8–12 spikes were generated by 500 ms current injections and the fast and medium AHPs were studied. Amplitude and duration of these AHP phases were quantified for the action potential closest to 250 ms after stimulus onset. In the *Fmr1* KO cell group, fast AHP amplitude (~28%) and duration (~55%) were reduced (Table 1); medium AHP amplitude decreased (~36%), while duration did not (Fig 4C, 4E and 4F; Table 1). Medium AHPs after similar repetitive firing were also studied. Some recorded cells of both groups showed an AHP following the action potential train. When present, the AHP amplitude was lower in the *Fmr1* KO cell group (Fig 4D; Table 1). These data support the notion that the absence of FMRP has a considerable impact on conductances underlying AHP.

## Discussion

The present study demonstrates an increase in excitability in hippocampal CA1 pyramidal cells from *Fmr1* KO mice compared with WT mice. The origin of this increase was not due to modifications in rheobase. The action potential duration was longer in cells from the *Fmr1* KO group, and the firing frequency evoked by current steps of the same intensity (100, 200, 300 and 400 pA) and duration (500 ms) were higher for this group. Moreover, the relationship between firing frequency and injected currents showed an almost 1.25-fold increase in the slope (gain) for cells from the *Fmr1* KO cell group in comparison with WT cells. Firing frequency increase of *Fmr1* KO cells was accompanied by a decrease in AHP amplitude. Modifications in input resistance, firing frequency gain and action potential duration reveal that the cell body input–output function is altered by the absence of FMRP in CA1 pyramidal neurons.

### Rheobase is not modified in CA1 pyramidal cells of *Fmr1* KO mice

The function of FMRP in altering membrane properties–excitability–from somatic recordings has previously been studied, and published reports suggest that FMRP-dependent intrinsic properties appear to be cell type-, brain region- and mouse strain-specific, as already proposed for dendritic properties [7, 17]. Membrane potential, input resistance, spike voltage threshold and rheobase were similar in layer 5 pyramidal neurons of the somatosensory cortex in WT and *Fmr1* KO mice [15, 18]. Layer 4 excitatory neurons of the somatosensory cortex exhibited a higher input resistance and lower rheoase in *Fmr1* KO mice; in contrast, no significant changes were seen in inhibitory neurons [19]. Two different populations of cells located in layer 5 of the medial prefrontal cortex have been studied in WT and *Fmr1* KO mice: pyramidal tract-projecting neurons and intratelencephalic-projecting neurons. Pyramidal tract-projecting neurons of the *Fmr1* KO mice showed a more hyperpolarized resting potential, similar input resistance and lower spike voltage threshold; in contrast to this population, the subthreshold properties of intratelencephalic-projecting neurons were similar in WT and *Fmr1* KO mice [17]. In the entorhinal cortex, a decreased action potential threshold was observed in layer II pyramidal cells of the *Fmr1* KO mice, while no changes were observed in stellate cell [21]. A previous study on CA1 pyramidal cells of the hippocampus reported that dendritic input resistance was lower in *Fmr1* KO mice versus WT and suggested that this alteration could be due in part to an enhancement of the hyperpolarization-activated non selective cationic current, I_h_ [13]. In addition, this study found no significant differences between WT and *Fmr1* KO mice using somatic recordings in input resistance and voltage sag. The present study, which was also performed on CA1 pyramidal cells, but in a different mouse strain, found a higher somatic input resistance (~ 15%) in the *Fmr1* KO cell group. Because we did not find significant differences in sag, a unique characteristic of h-channels, and resting membrane potential was not more hyperpolarized, higher somatic input resistance in *Fmr1* KO cells cannot reliably be attributed to a lower I_h_. To support this proposal other studies would be needed (for example, subthreshold electrical resonance in the theta frequency range, the impact of ZD7288 on I_h_ membrane properties, quantification of I_h_) [13, 17]. On the other hand, membrane properties (resting membrane potential, input resistance and action potential threshold) play a major role in setting neuronal excitability. These membrane properties are dependent on tonic excitatory/inhibitory inputs and /or on subthreshold currents such as M-current, H-current, I_NaP_ [21] and potassium currents mediated by Kv1 and Kv4 channels [17]. Determining how alterations in synaptic inputs and /or subthreshold membrane currents contribute to increased input resistance in CA1 pyramidal cells of *Fmr1* KO mice requires further investigation.

We have reported here an increased input resistance in CA1 pyramidal cells of *Fmr1* KO mice and no significant changes in resting membrane potential, action potential threshold, or rheobase between groups. Taken into account these data, what could the functional consequences of an increased input resistance in the *Fmr1* KO cell group be? Input resistance changes could result in modification in cell excitability and /or could occlude normal homeostatic intrinsic plasticity in this parameter that occurs during long term synaptic plasticity [13, 14, 15, 17]. Input resistance and rheobase are often inversely related. Significant changes in rheobase shift to the right or left the relationship between action potential firing frequency versus current intensity, leading to an increase or decrease in cell excitability. Depolarizing current steps are widely used to determine rheobase. However, depolarizing current steps do not exactly replicate either the spatial and temporal summation of excitatory/inhibitory postsynaptic currents or accompanying membrane potential fluctuations which underlie the probability of a neuron eliciting an action potential [31, 32]. For instance, an increased input resistance and longer membrane time constant, mediated by a reduction in M-current, augment the magnitude of excitatory postsynaptic potentials, as well as temporal and spatial summation of subthreshold potentials in hippocampal CA1 pyramidal cells [35]. In accord with these notions, an increased input resistance in *Fmr1* KO cells could increase cell excitability, even though rheobase did not decrease.

With regards to input resistance plasticity, it was found that transient and persistent changes in input resistance—I_h_ occur during and after induction of synaptic plasticity [36, 37]. Brager and colleagues [13] reported that the activation of group I metabotropic glutamate receptors results in long-term depression (LTD) and an increased input resistance in CA1 pyramidal neurons in both WT and *Fmr1* KO mice. In addition, they also found that a theta-burst firing of action potentials paired with excitatory postsynaptic potentials (TBP) resulted in long term potentiation (LTP) which was accompanied by a significant decrease in input resistance in WT mice, but not in *Fmr1* KO mice. These changes in input resistance were in part attributed to modifications in Ih. According to these data they pointed out that elevation in dendritic Ih (that is, lower input resistance) appears to occlude normal homeostatic intrinsic plasticity of Ih—input resistance that occurs during TBP-LTP in *Fmr1* KO mice [13]. Taking into account these findings and that we have reported here a higher input resistance at the soma of *Fmr1* KO mice while Brager and colleagues did not [13], a replication of their experiments should be carried out to investigate whether a higher somatic input resistance in *Fmr1* KO mice occludes the normal homeostatic intrinsic plasticity in this parameter with the activation of group I metabotropic glutamate receptors.

### Action potential duration is increased in CA1 pyramidal cells of *Fmr1* KO mice

Deng and colleagues [23] studied the effect of FMRP on action potential duration in pyramidal cells of the hippocampal CA3 region and layer 5 of the entorhinal cortex. They reported two different cell populations in the entorhinal cortex. In one, excessive action potential broadening due to the absence of FMRP was similar to that in the hippocampal CA3 pyramidal cells (~130% of the baseline), whereas another population exhibited greatly exaggerated broadening (up to 300% of baseline) at the end of the train. On the other hand, they also demonstrated that FMRP regulates the action potential duration by altering the Ca^2+^ sensitivity of BK channels, and that elevated action potential broadening during a train of stimulation resulted in enhanced Ca^2+^ influx into presynaptic terminals. The present data show that an absence of FMRP gives rise to increased action potential duration in CA1 pyramidal cells that is of a similar magnitude to that reported for pyramidal cells in the CA3 hippocampal region [23]. In addition, somatic recordings from cortical neurons also identified action potentials of heightened duration in *Fmr1* KO mice [15]. Taken together, these findings suggest that FMRP acts via BK channels in the hippocampus and other brain regions, and that the functional alteration of these channels may result in an abnormally high neurotransmitter release contributing to the synaptic alteration in FXS.

### The slope of the firing frequency–current intensity relationship increases in CA1 pyramidal cells of *Fmr1* KO mice

Few studies of WT and *Fmr1* KO mice have compared action potential firing frequencies in response to current injections. Neither the firing frequency–current injection relationship, nor the spike frequency adaption showed any abnormality in pyramidal neurons located in layer 5 of the somatosensory cortex in *Fmr1* KO mice [18]. In the absence of FMRP, excitatory neurons in layer 4 of the somatosensory cortex fired more action potentials for a given 600 ms current injection, with this increased excitability attributed, in part, to the increased input resistance [19]. The number of action potentials evoked as a function of current injected and the action potential rate gain were slightly increased in layer 5 *Fmr1* KO neurons in the somatosensory cortex [15]. The present data obtained from CA1 pyramidal cells in *Fmr1* KO mice showed a higher number of action potentials for current steps of 100, 200, 300 and 400 pA and the slope of the firing frequency–current injection relationship increased 1.25-fold.

The increased firing frequency gain in CA1 pyramidal cells from the *Fmr1 KO* mice could have its origin in changes in ionic currents underlying action potential shape, ADP and/or AHP [22, 38, 39, 40]. A recent study carried out in layer 2/3 prefrontal cortex neurons reported that the gain of action potential output in response to DC current injection was increased in *Fmr1 KO* neurons [22]. In addition, action potentials were larger, faster and narrower. These latter properties underlie larger transient Na+ current and activation curve of somatic A-type K+ current that was depolarized. According to neuronal simulations, these biophysical changes could be sufficient to explain the increase in action potential firing. The increased firing frequency gain in CA1 pyramidal cells from *Fmr1 KO* mice reported here cannot be reliably supported by these mechanisms, since action potential had longer duration and shorter amplitude. In CA1 pyramidal cells the amplitude of ADP depends on a balance between the depolarizing persistent Na^+^ current and hyperpolarizing M current (Kv 7/ M channels); when M-current is blocked, the neuron remains depolarized for a lengthy period during which it may generate multiple spikes [38]. The AHP features determine the refractoriness and firing frequency. We have reported here lower amplitudes of both fast- and medium-AHP. Lower amplitude of fast AHP could result from a reduced conductance via BK channels [23]. The medium AHP plays a major role to determine firing frequency and, at least, three different types of ion channels may be active during this period in CA1 pyramidal cells: SK-, HCN- and Kv7/M-channels. Studies on SK channels have concluded that these channels contribute little or nothing to the somatic mAHP [39, 41]; but, when Kv7/M channel activity is compromised, SK channel activation significantly limits spike discharge [42]. Kv7/M- and HCN-channels generate somatic mAHP and their contribution is related to membrane potential [38]. Interestingly, inhibition of Kv7/M channels by XE991 [39, 40] and the absence of FMRP (present results) in CA1 pyramidal cells produce some similar effects: i) the input resistance increases; ii) medium AHP is abolished if a single action potential is evoked by drifting the membrane potential to spike threshold; iii) the amplitude and duration of the medium AHP are reduced after a train of action potentials; and iv) in response to suprathreshold depolarizing current steps of the same magnitude, the number of spikes increases, whereas the amplitude of the medium AHP decreases. However, the blockage of the Kv7/ M channel also produces some effects not found here: i) resting membrane potential is more depolarized; ii) the ADP is enhanced and promoted spike bursting; iii) a lower firing frequency adaptation [38, 39]. On the other hand, *Fmr1* KO cells show an action potential broadening which elevates calcium influx [23]. A rise of intracellular calcium reduces M current [43] which, in turn, increase ADP amplitude [15] and decrease medium AHP amplitude [38]. According to these studies, the contribution of intracellular calcium to shape ADP and medium AHP, as well as to control firing properties in *Fmr1* KO cells would require further research. Therefore, FMRP absence could increase firing frequency gain by a diminished activity of the Kv7/M channels and /or by dysfunction of other ion channels that also underlie somatic spike afterpotentials.

Synaptic dysfunction could also contribute to increased firing frequency gain in CA1 pyramidal cells from *Fmr1 KO* mice [44–49]. Because the primary goal of this work was to determine whether FMRP absence generates an increase in intrinsic excitability independent of its origin, we did not block synaptic receptors. Studies on change in cell excitability associated with synaptic dysfunctions in *Fmr1* KO mice are scarce. In absence of FMRP epileptogenesis produced by the activation of group I metabotropic glutamate receptor (mGluR) is enhanced in CA3 pyramidal cells [44]; indeed, FMRP acts as a brake on group I mGluR-mediated translation and epileptogenesis [45]. Hyperexcitability in *Fmr1* KO mice may also be explained by decreases in inhibitory transmission which can depend on: i) endocannabinoids mobilization mediated by activation of group I mGluR [46, 47]; ii) reduced phasic and tonic GABAergic currents mediated by ionotropic receptors [48]; and iii) presynaptic GABA_B_ receptor-dependent reduction in GABA release [49]. We have no evidence to establish any causal relationship between changes in the input-ouput function reported in this study and the above-mentioned glutamatergic and/or GABAergic disorders in *Fmr1* KO mice. As a consequence, more research in this field is recommended.

### Functional implications

The present study has demonstrated that the action potential duration was longer and firing frequency gain was higher in the CA1 pyramidal cells of *Fmr1* KO mice compared with WT mice. These biophysical changes produce an increase in the excitability of CA1 pyramidal cell body in *Fmr1* KO mice, which would lead to increased neurotransmitter release. Knowledge of the cellular- and synaptic-mechanisms underlying cell body hyperexcitability produced by the FMRP absence is relevant to better understand the clinical features of FXS and could provide a potentially rich source of pharmacological and genetic therapies for treatment of this neurological disorder.

## Supporting information

S1 Table Membrane properties of the CA1 pyramidal cells in wild type mice. (PDF)

S2 Table Membrane properties of the CA1 pyramidal cells in *Fmr1* KO mice. (PDF)

## References

[1] Pfeiffer BE, Huber KM. The state of synapses in fragile X syndrome. Neuroscientist 2009; 15: 549–567. doi: 10.1177/1073858409333075 19325170 10.1177/1073858409333075 PMC2762019

[2] Bear MF, Huber KM, Warren ST. The mGluR theory of fragile X mental retardation. Trends Neurosci 2004; 27: 370–377. doi: 10.1016/j.tins.2004.04.009 15219735 10.1016/j.tins.2004.04.009

[3] Penagarikano O, Mulle JG, Warren ST. The pathophysiology of fragile x syndrome. Annu Rev Genomics Hum Genet 2007; 8:109–129. doi: 10.1146/annurev.genom.8.080706.092249 17477822 10.1146/annurev.genom.8.080706.092249

[4] Bassell GJ, Warren ST. Fragile X syndrome: loss of local mRNA regulation alters synaptic development and function. Neuron 2008; 60: 201–214. doi: 10.1016/j.neuron.2008.10.004 18957214 10.1016/j.neuron.2008.10.004 PMC3691995

[5] Brager DH, Johnston D. Channelopathies and dendritic dysfunction in fragile X syndrome. Brain Res Bull 2014; 103:11–17. doi: 10.1016/j.brainresbull.2014.01.002 24462643 10.1016/j.brainresbull.2014.01.002 PMC4049233

[6] Santos AR, Kanellopoulos AK, Bagni C. Learning and behavioral deficits associated with the absence of the fragile X mental retardation protein: what a fly and mouse model can teach us. Learn Mem 2014; 21:543–55. doi: 10.1101/lm.035956.114 25227249 10.1101/lm.035956.114 PMC4175497

[7] Contractor A, Klyachko VA, Portera-Cailliau C. Altered Neuronal and Circuit Excitability in Fragile X Syndrome. Neuron 2015; 87: 699–715. doi: 10.1016/j.neuron.2015.06.017 26291156 10.1016/j.neuron.2015.06.017 PMC4545495

[8] Huber KM, Gallagher SM, Warren ST, Bear MF. Altered synaptic plasticity in a mouse model of fragile X mental retardation. Proc Natl Acad Sci USA 2002; 99: 7746–7750. doi: 10.1073/pnas.122205699 12032354 10.1073/pnas.122205699 PMC124340

[9] Li J, Pelletier MR, Perez Velazquez JL, Carlen PL. Reduced cortical synaptic plasticity and GluR1 expression associated with fragile X mental retardation protein deficiency. Mol Cell Neurosci 2002; 19: 138–151. doi: 10.1006/mcne.2001.1085 11860268 10.1006/mcne.2001.1085

[10] Deng PY, Sojka D, Klyachko VA. Abnormal presynaptic short-term plasticity and information processing in a mouse model of fragile X syndrome. J Neurosci 2011; 31:10971–10982. doi: 10.1523/JNEUROSCI.2021-11.2011 21795546 10.1523/JNEUROSCI.2021-11.2011 PMC6623101

[11] Gross C, Yao X, Pong DL, Jeromin A, Bassell GJ. Fragile X mental retardation protein regulates protein expression and mRNA translation of the potassium channel Kv4.2. J Neurosci 2011; 31: 5693–5698. doi: 10.1523/JNEUROSCI.6661-10.2011 21490210 10.1523/JNEUROSCI.6661-10.2011 PMC3089949

[12] Lee HY, Ge WP, Huang W, He Y, Wang GX, Rowson-Baldwin A et al Bidirectional regulation of dendritic voltage-gated potassium channels by the fragile X mental retardation protein. Neuron 2011; 72: 630–642. doi: 10.1016/j.neuron.2011.09.033 22099464 10.1016/j.neuron.2011.09.033 PMC3433402

[13] Brager DH, Akhavan AR, Johnston D. Impaired dendritic expression and plasticity of h-channels in the fmr1(-/y) mouse model of fragile X syndrome. Cell Rep 2012; 1: 225–233. doi: 10.1016/j.celrep.2012.02.002 22662315 10.1016/j.celrep.2012.02.002 PMC3363364

[14] Routh BN, Johnston D, Brager DH. Loss of functional A-type potassium channels in the dendrites of CA1 pyramidal neurons from a mouse model of fragile X syndrome. J Neurosci 2013; 33: 19442–19450. doi: 10.1523/JNEUROSCI.3256-13.2013 24336711 10.1523/JNEUROSCI.3256-13.2013 PMC3858620

[15] Zhang Y, Bonnan A, Bony G, Ferezou I, Pietropaolo S, Ginger M et al Dendritic channelopathies contribute to neocortical and sensory hyperexcitability in Fmr1(-/y) mice. Nat Neurosci 2014; 17: 1701–1719. doi: 10.1038/nn.3864 25383903 10.1038/nn.3864

[16] Ferron L, Nieto-Rostro M, Cassidy JS, Dolphin AC. Fragile X mental retardation protein controls synaptic vesicle exocytosis by modulating N-type calcium channel density. Nat Commun 2014; 5: 3628 doi: 10.1038/ncomms4628 24709664 10.1038/ncomms4628 PMC3982139

[17] Kalmbach BE, Johnston D, Brager DH. Cell-Type Specific Channelopathies in the Prefrontal Cortex of the fmr1-/y Mouse Model of Fragile X Syndrome. 2015; (1,2,3).eNeuro.2(6). pii: ENEURO.0114-15. 10.1523/ENEURO.0114-15.2015 PMC4647062 26601124

[18] Desai NS, Casimiro TM, Gruber SM, Vanderklish PW. Early postnatal plasticity in neocortex of Fmr1 knockout mice. J Neurophysiol 2006; 96: 1734–1745. doi: 10.1152/jn.00221.2006 16823030 10.1152/jn.00221.2006

[19] Gibson JR, Bartley AF, Hays SA, Huber KM. Imbalance of neocortical excitation and inhibition and altered UP states reflect network hyperexcitability in the mouse model of fragile X syndrome. J Neurophysiol 2008; 100: 2615–2626. doi: 10.1152/jn.90752.2008 18784272 10.1152/jn.90752.2008 PMC2585391

[20] Zhang L, Liang Z, Zhu P, Li M, Yi YH, Liao WP et al Altered intrinsic properties and bursting activities of neurons in layer IV of somatosensory cortex from *Fmr-1* knockout mice. Exp Neurol 2016; 280: 60–69 doi: 10.1016/j.expneurol.2016.03.025 27048919 10.1016/j.expneurol.2016.03.025

[21] Deng PY, Klyachko VA. Increased Persistent Sodium Current Causes Neuronal Hyperexcitability in the Entorhinal Cortex of Fmr1 Knockout Mice. Cell Rep 2016; 16: 3157–3166 doi: 10.1016/j.celrep.2016.08.046 27653682 10.1016/j.celrep.2016.08.046 PMC5055130

[22] Routh BN, Rathour RK, Baumgardner ME, Kalmbach BE, Johnston D, Brager DH. Increased transient Na+ conductance and action potential output in layer 2/3 prefrontal cortex neurons of the fmr1-/y mouse. J Physiol 2017; 595: 4431–4448 doi: 10.1113/JP274258 28370141 10.1113/JP274258 PMC5491866

[23] Deng PY, Rotman Z, Blundon JA, Cho Y, Cui J, Cavalli V et al FMRP regulates neurotransmitter release and synaptic information transmission by modulating action potential duration via BK channels. Neuron 2013; 77: 696–711. doi: 10.1016/j.neuron.2012.12.018 23439122 10.1016/j.neuron.2012.12.018 PMC3584349

[24] Christie SB, Akins MR, Schwob JE, Fallon JR. The FXG: a presynaptic fragile X granule expressed in a subset of developing brain circuits. J Neurosci 2009; 29: 1514–1524. doi: 10.1523/JNEUROSCI.3937-08.2009 19193898 10.1523/JNEUROSCI.3937-08.2009 PMC2746438

[25] Brown V, Jin P, Ceman S, Darnell JC, O'Donnell WT, Tenenbaum SA, Jin X, Feng Y, Wilkinson KD, Keene JD, Darnell RB, Warren ST. Microarray identification of FMRP-associated brain mRNAs and altered mRNA translational profiles in fragile X syndrome. Cell 2001; 107: 477–487. 11719188 10.1016/s0092-8674(01)00568-2

[26] Brown MR, Kronengold J, Gazula VR, Chen Y, Strumbos JG, Sigworth FJ et al Fragile X mental retardation protein controls gating of the sodium-activated potassium channel Slack. Nat Neurosci 2010; 13: 819–821. doi: 10.1038/nn.2563 20512134 10.1038/nn.2563 PMC2893252

[27] Darnell JC, Van Driesche SJ, Zhang C, Hung KY, Mele A, Fraser CE et al FMRP stalls ribosomal translocation on mRNAs linked to synaptic function and autism. Cell 2011; 146: 247–261. doi: 10.1016/j.cell.2011.06.013 21784246 10.1016/j.cell.2011.06.013 PMC3232425

[28] Zhang Y, Brown MR, Hyland C, Chen Y, Kronengold J, Fleming MR et al Regulation of neuronal excitability by interaction of fragile X mental retardation protein with slack potassium channels. J Neurosci 2012; 32: 15318–15327. doi: 10.1523/JNEUROSCI.2162-12.2012 23115170 10.1523/JNEUROSCI.2162-12.2012 PMC3518385

[29] Myrick LK, Deng PY, Hashimoto H, Oh YM, Cho Y, Poidevin MJ et al Independent role for presynaptic FMRP revealed by an FMR1 missense mutation associated with intellectual disability and seizures. Proc Natl Acad Sci USA 2015; 112: 949–956. doi: 10.1073/pnas.1423094112 25561520 10.1073/pnas.1423094112 PMC4313821

[30] Beck H, Yaari Y. Plasticity of intrinsic neuronal properties in CNS disorders. Nat Rev Neurosci 2008; 9: 357–369. doi: 10.1038/nrn2371 18425090 10.1038/nrn2371

[31] Mitchell SJ, Silver RA. Shunting inhibition modulates neuronal gain during synaptic excitation. Neuron 2003; 38: 433–445. 12741990 10.1016/s0896-6273(03)00200-9

[32] Carvalho TP, Buonomano DV. Differential effects of excitatory and inhibitory plasticity on synaptically driven neuronal input-output functions. Neuron 2009; 61: 774–785. doi: 10.1016/j.neuron.2009.01.013 19285473 10.1016/j.neuron.2009.01.013 PMC2676350

[33] Paxinos G, Franklin KBJ. The mouse brain in stereotaxic coordinates San Diego: Academic Press; 2001 p

[34] Storm JF. Action potential repolarization and a fast after-hyperpolarization in rat hippocampal pyramidal cells. J Physiol 1987; 385: 733–759. 2443676 10.1113/jphysiol.1987.sp016517 PMC1192370

[35] Hönigsperger C, Marosi M, Murphy R, Storm JF. Dorsoventral differences in Kv7/M-current and its impact on resonance, temporal summation and excitability in rat hippocampal pyramidal cells. J Physiol 2015, 593; 1551–1580 doi: 10.1113/jphysiol.2014.280826 25656084 10.1113/jphysiol.2014.280826 PMC4386960

[36] Fan Y, Fricker D, Brager DH, Chen X, Lu HC, Chitwood RA et al Activity-dependent decrease of excitability in rat hippocampal neurons through increases in I(h). Nat Neurosci 2005, 8; 1542–1551 doi: 10.1038/nn1568 16234810 10.1038/nn1568

[37] Brager DH, Johnston D. Plasticity of intrinsic excitability during long-term depression is mediated through mGluR-dependent changes in I(h) in hippocampal CA1 pyramidal neurons. J Neurosci 2007, 27; 13926–13937 doi: 10.1523/JNEUROSCI.3520-07.2007 18094230 10.1523/JNEUROSCI.3520-07.2007 PMC6673524

[38] Yue C, Yaari Y. KCNQ/M channels control spike afterdepolarization and burst generation in hippocampal neurons. J Neurosci 2004; 12: 4614–4626 10.1523/JNEUROSCI.0765-04.2004 PMC6729392 15140933

[39] Gu N, Vervaeke K, Hu H, Storm JF. Kv7/KCNQ/M and HCN/h, but not KCa2/SK channels, contribute to the somatic medium after-hyperpolarization and excitability control in CA1 hippocampal pyramidal cells. J Physiol 2005; 566: 689–715. doi: 10.1113/jphysiol.2005.086835 15890705 10.1113/jphysiol.2005.086835 PMC1464792

[40] Peters HC, Hu H, Pongs O, Storm JF, Isbrandt D. Conditional transgenic suppression of M channels in mouse brain reveals functions in neuronal excitability, resonance and behavior. Nat Neurosci 2005; 8: 51–60. doi: 10.1038/nn1375 15608631 10.1038/nn1375

[41] Gu N, Hu H, Vervaeke K, Storm JF. SK (KCa2) channels do not control somatic excitability in CA1 pyramidal neurons but can be activated by dendritic excitatory synapses and regulate their impact. J Neurophysiol 2008; 100: 2589–2604. doi: 10.1152/jn.90433.2008 18684909 10.1152/jn.90433.2008

[42] Chen S, Benninger F, Yaari Y. Role of small conductance Ca^2+^-activated K^+^ channels in controlling CA1 pyramidal cell excitability. J Neurosci 2014; 34: 8219–8230. doi: 10.1523/JNEUROSCI.0936-14.2014 24920626 10.1523/JNEUROSCI.0936-14.2014 PMC6608239

[43] Selyanko AA, Brown DA. Intracellular calcium directly inhibits potassium M channels in excised membrane patches from rat sympathetic neurons. Neuron 1996, 16; 151–162 8562079 10.1016/s0896-6273(00)80032-x

[44] Bianchi R, Chuang SC, Zhao W, Young SR, Wong RK. Cellular plasticity for group I mGluR-mediated epileptogenesis. J Neurosci 2009; 29: 3497–3507. doi: 10.1523/JNEUROSCI.5447-08.2009 19295155 10.1523/JNEUROSCI.5447-08.2009 PMC2692254

[45] Zhao W, Chuang SC, Bianchi R, Wong RK. Dual regulation of fragile X mental retardation protein by group I metabotropic glutamate receptors controls translation-dependent epileptogenesis in the hippocampus. J Neurosci 2011; 31:725–734. doi: 10.1523/JNEUROSCI.2915-10.2011 21228181 10.1523/JNEUROSCI.2915-10.2011 PMC6623451

[46] Zhang L, Alger BE. Enhanced endocannabinoid signaling elevates neuronal excitability in fragile X syndrome. J Neurosci 2010; 30: 5724–5729 doi: 10.1523/JNEUROSCI.0795-10.2010 20410124 10.1523/JNEUROSCI.0795-10.2010 PMC2906112

[47] Tang AH and Alger BE. Homer protein-metabotropic glutamate receptor binding regulates endocannabinoid signaling and affects hyperexcitability in a mouse model of fragile X syndrome. J Neurosci 2015; 35: 3938–3945. doi: 10.1523/JNEUROSCI.4499-14.2015 25740522 10.1523/JNEUROSCI.4499-14.2015 PMC4348189

[48] Olmos-Serrano JL, Paluszkiewicz SM, Martin BS, Kaufmann WE, Corbin JG, Huntsman MM. Defective GABAergic neurotransmission and pharmacological rescue of neuronal hyperexcitability in the amygdala in a mouse model of fragile X syndrome. J Neurosci 2010; 30: 9929–9938. doi: 10.1523/JNEUROSCI.1714-10.2010 20660275 10.1523/JNEUROSCI.1714-10.2010 PMC2948869

[49] Wahlstrom-Helgren S and Klyachko VA. GABA_B_ receptor-mediated feed-forward circuit dysfunction in the mouse model of fragile X syndrome. J Physiol 2015; 593: 5009–5024. doi: 10.1113/JP271190 26282581 10.1113/JP271190 PMC4650406

