# Supplementary material for: Excitability is increased in hippocampal CA1 pyramidal cells of Fmr1 knockout mice
Source: PLoS One. 2017 Sep 20;12(9):e0185067. doi: 10.1371/journal.pone.0185067 (PMC5607184; doi:10.1371/journal.pone.0185067)
Supplement: S1 Table — (PDF) [file pone.0185067.s001.pdf]

Membrane properties of the CA1 pyramidal cells in wild type mice						
	**cell 1**	**cell 2**	**cell 3**	**cell 4**	**cell 5**	**cell 6**
Resting membrane potential (mV)	-61	-62.9	-67.9	-64	-66.5	-60.3
Input resistance (MΩ)	198.3	181.8	144.4	191.4	106.2	187.5
Sag (%)		14.7	20.1	12.78	16.24	14.21
Time constant (ms)	14.93	17.32	12.82	18	13.99	20.43
Rheobase (pA)						
Voltage threshold (mV)						
Depolarization voltage (mV)						
Amplitude action potential (mV)	98.4	115.02	121.03	115.42	124.45	102.83
Action potential duration (ms)						
Single action potential	0.95	0.92	0.93	1.05	0.97	0.83
Firing train (8-12 AP during 500 ms)						
First						
Middle						
Last						
Firing frequency (AP x s-1)						
100 pA						
200 pA						
300 pA						
400 pA						
F/I gain						
ADP/AHP						
ADP single action potential						
Amplitude	6.59	4.12	10.1	12.57	11.93	12.97
Duration	51.4	29.8	60.3	89.6	100.6	23.6
AHP low frequency (<2 Ap x s-1)						
fAHP Amplitude (mV)						
fAHP Duration (ms)						
mAHP Amplitude (mV)						
mAHP Duration (ms)						
AHP firing train (8-12 AP during 500 ms)						
fAHP Amplitude (mV)						
fAHP Duration (ms)						
mAHP Amplitude (mV)						
mAHP Duration (ms)						
mAHP after a firing train (8-12 AP during 500 ms)						
Amplitude (mV)						
Duration (ms)						
Adaptation index						

Membrane properties of the CA1 pyramidal cells in wild type mice						
	**cell 7**	**cell 8**	**cell 9**	**cell 10**	**cell 11**	**cell 12**
Resting membrane potential (mV)	-67.5	-62.5	-66.5	-62.5	-64	-59
Input resistance (MΩ)	195.8	124.8	126.9	131.3	96.87	93.12
Sag (%)	16.29	20.56	17.14	17.2	20.61	13.94
Time constant (ms)	22.7	25.01	18.96	18.96	21.44	19.86
Rheobase (pA)			130		150	30
Voltage threshold (mV)			-41.84		-45.07	-43.32
Depolarization voltage (mV)			24.51		19.87	15.68
Amplitude action potential (mV)	114.75	122.03	123	113.8	123.52	114.35
Action potential duration (ms)						
Single action potential	0.97	0.85	0.85	0.95	0.92	0.92
Firing train (8-12 AP during 500 ms)						
First	1.01	0.87	0.9	0.97		0.84
Middle	1.01	0.91	0.95	1.01		0.89
Last	1.01	0.92	0.99	1.01		0.91
Firing frequency (AP x s-1)						
100 pA	20	12	0	24		4
200 pA	28	28	16	36		12
300 pA	28	40	28	48		20
400 pA		48	36	52		24
F/I gain	0.09	0.15	0.13	0.16		0.08
ADP/AHP						
ADP single action potential						
Amplitude	11.44	7.05	7.72	4.3	10.07	1.74
Duration	47.95	56.4	57.15	75.35	62.75	53.8
AHP low frequency (<2 Ap x s-1)						
fAHP Amplitude (mV)			12.68		11.43	9.34
fAHP Duration (ms)			2.67		4.8	3.95
mAHP Amplitude (mV)			11.88		9.2	8.6
mAHP Duration (ms)			189.07		211.15	166.95
AHP firing train (8-12 AP during 500 ms)						
fAHP Amplitude (mV)	3.63	10.26	12.5	11.17		9.67
fAHP Duration (ms)	2.35	1.55	1.57	3.1		2.71
mAHP Amplitude (mV)	4.15	9.5	11.65	5.95		6.17
mAHP Duration (ms)	69.7	51.35	64.25	60.5		71.73
mAHP after a firing train (8-12 AP during 500 ms)						
Amplitude (mV)	2.53	2.32	1.62			
Duration (ms)	80.15	111.65	98.45			
Adaptation index	0.59	0.54	0.35	0.1		0.69

Membrane properties of the CA1 pyramidal cells in wild type mice						
	**cell 13**	**cell 14**	**cell 15**	**cell 16**	**cell 17**	**cell 18**
Resting membrane potential (mV)	-66	-63.5	-60.7	-60	-61	-61.5
Input resistance (MΩ)	168.1		207.6	202.4	94	163
Sag (%)	14.1			15.54		
Time constant (ms)	15.34		29.53	21.37	18.83	15.28
Rheobase (pA)	104	210	26	26		60
Voltage threshold (mV)	-42.48	-39.09	-51.33	-50.17		-50.7
Depolarization voltage (mV)	23.83	25.09	8.94	10.1		10.89
Amplitude action potential (mV)			111.8		118.85	116.49
Action potential duration (ms)						
Single action potential			1.18		1	1.13
Firing train (8-12 AP during 500 ms)						
First		1		1		
Middle		1.03		1.04		
Last		1.03		1.02		
Firing frequency (AP x s-1)						
100 pA	2	0		24	12	0
200 pA	20	4		40	24	4
300 pA	32	16		48	32	16
400 pA	32	24		56	40	12
F/I gain	0.14	0.12		0.17	0.13	0.07
ADP/AHP						
ADP single action potential						
Amplitude			2.41		5.65	
Duration			81.45		72.6	
AHP low frequency (<2 Ap x s-1)						
fAHP Amplitude (mV)	7.63	9.3	6.96	12.18		13.47
fAHP Duration (ms)	9	2.76	1.5	1.87		5.88
mAHP Amplitude (mV)	8.1	8.4	4	10.75		13.49
mAHP Duration (ms)	158.5	108.24	118.55	192.6		115.2
AHP firing train (8-12 AP during 500 ms)						
fAHP Amplitude (mV)		8.83		10.99		
fAHP Duration (ms)		2.35		2.65		
mAHP Amplitude (mV)		6.385		9.04		
mAHP Duration (ms)		50		43.2		
mAHP after a firing train (8-12 AP during 500 ms)						
Amplitude (mV)						
Duration (ms)						
Adaptation index		0.31		0.17		

**Membrane properties of the CA1 pyramidal cells in wild type mice**						
	**cell 19**	**cell 20**	**cell 21**	**cell 22**	**cell 23**	**cell 24**
Resting membrane potential (mV)	-60.5	-70	-60.5	-63.4	-67	-61.4
Input resistance (MΩ)	150	100	197	135	178	190
Sag (%)						
Time constant (ms)	29.2	25.69	18.13	20.69	23.13	24.73
Rheobase (pA)		200	56	42	134	24
Voltage threshold (mV)		-49.7	-42.3	-51.8	-49.4	-48.3
Depolarization voltage (mV)		22.74	16.24	9.61	18.79	10.44
Amplitude action potential (mV)		131.1		117.16	121.93	113.89
Action potential duration (ms)						
Single action potential		1		1.04	1.12	0.91
Firing train (8-12 AP during 500 ms)						
First						0.9
Middle						0.9
Last						0.92
Firing frequency (AP x s-1)						
100 pA	4		12	12	8	16
200 pA	24		20	28	28	20
300 pA	32		28	40	40	36
400 pA	40		32	44	40	44
F/I gain	0.13		0.1	0.14	0.16	0.12
ADP/AHP						
ADP single action potential						
Amplitude		6.5		6.29	6.96	
Duration		52.5		73.65	114.25	
AHP low frequency (<2 Ap x s-1)						
fAHP Amplitude (mV)		11.58	7.3	4.84	10.61	19.61
fAHP Duration (ms)		3.57	1.79	1.62	3.15	3.85
mAHP Amplitude (mV)		11.32	6.96	4.3	10.5	12.66
mAHP Duration (ms)		102.7	176.75	130.7	163.15	210
AHP firing train (8-12 AP during 500 ms)						
fAHP Amplitude (mV)						20.72
fAHP Duration (ms)						1.3
mAHP Amplitude (mV)						21.21
mAHP Duration (ms)						59.97
mAHP after a firing train (8-12 AP during 500 ms)						
Amplitude (mV)						
Duration (ms)						
Adaptation index						0.22

Membrane properties of the CA1 pyramidal cells in wild type mice						
	**cell 25**	**cell 26**	**cell 27**	**cell 28**	**cell 29**	**cell 30**
Resting membrane potential (mV)	-60.8	-68.1	-60.5	-60.9	-64.4	-63.6
Input resistance (MΩ)	82	160	198	140	140	196
Sag (%)						
Time constant (ms)	17.81	16.26	22.58	20.1	22.39	24.3
Rheobase (pA)	62	44	50	46	80	60
Voltage threshold (mV)	-52.7	-57.2	-43.7	-44.7	-49.3	-41.1
Depolarization voltage (mV)	5.37	12.12	16.75	14.13	13.82	21.48
Amplitude action potential (mV)	118.43	125.97	118.23	115.78	124.47	122.54
Action potential duration (ms)						
Single action potential	1.06	0.98	1.1	1.1	1.07	1.06
Firing train (8-12 AP during 500 ms)						
First	1.05	0.93				
Middle	1.05	0.97				
Last	1.05	0.98				
Firing frequency (AP x s-1)						
100 pA	0	16	24	8		12
200 pA	8	20	36	24		28
300 pA	16	36	48	32		
400 pA	32	44	56	36		
F/I gain	0.04	0.11	0.15	0.12		0.14
ADP/AHP						
ADP single action potential						
Amplitude	4.21	8.54	12.27		9.8	10.04
Duration	61.3	64.35	68.8		59.7	70.25
AHP low frequency (<2 Ap x s-1)						
fAHP Amplitude (mV)	4.43	7.25	8.81	16.63	9.47	18.28
fAHP Duration (ms)	1.74	1.08	3.15	7.4	3	3.1
mAHP Amplitude (mV)	4.27	3.39	3.27	12.12	4.67	6.56
mAHP Duration (ms)	87.5	190.8	219.55	261.55	183.4	213.5
AHP firing train (8-12 AP during 500 ms)						
fAHP Amplitude (mV)	8.94	10.1				
fAHP Duration (ms)	1.75	1.15				
mAHP Amplitude (mV)	10.71	10.59				
mAHP Duration (ms)	59.25	71.6				
mAHP after a firing train (8-12 AP during 500 ms)						
Amplitude (mV)		2.32				
Duration (ms)		170.6				
Adaptation index	0.72	0.73				

**Membrane properties of the CA1 pyramidal cells in wild type mice**			
	**cell 31**	**cell 32**	**cell 33**
Resting membrane potential (mV)	-65.9	-64	-61.5
Input resistance (MΩ)	122	152	104
Sag (%)			
Time constant (ms)	12.86	27.55	23.07
Rheobase (pA)	44	70	38
Voltage threshold (mV)	-51.6	-45.7	-56.2
Depolarization voltage (mV)	11.66	18.43	4.73
Amplitude action potential (mV)	126.32	123.47	103.96
Action potential duration (ms)			
Single action potential	1.03	1.03	1.05
Firing train (8-12 AP during 500 ms)			
First		1.02	1.05
Middle		1.02	1.02
Last		1.02	1.02
Firing frequency (AP x s-1)			
100 pA		8	16
200 pA		20	28
300 pA		32	36
400 pA		40	44
F/I gain		0.13	0.12
ADP/AHP			
ADP single action potential			
Amplitude	10.16	8.73	
Duration	85.55	69.85	
AHP low frequency (<2 Ap x s-1)			
fAHP Amplitude (mV)	5.97	14.58	4.09
fAHP Duration (ms)	2.05	3.3	2.12
mAHP Amplitude (mV)	1.34	7.29	3.62
mAHP Duration (ms)	205.25	154.75	129.55
AHP firing train (8-12 AP during 500 ms)			
fAHP Amplitude (mV)		14.34	6.87
fAHP Duration (ms)		2.8	1.35
mAHP Amplitude (mV)		13.61	6.76
mAHP Duration (ms)		43.55	54.3
mAHP after a firing train (8-12 AP during 500 ms)			
Amplitude (mV)		1.98	
Duration (ms)		145.1	
Adaptation index		0.46	0.67
