# Supplementary material for: Excitability is increased in hippocampal CA1 pyramidal cells of Fmr1 knockout mice
Source: PLoS One. 2017 Sep 20;12(9):e0185067. doi: 10.1371/journal.pone.0185067 (PMC5607184; doi:10.1371/journal.pone.0185067)
Supplement: S2 Table — (PDF) [file pone.0185067.s002.pdf]

Membrane properties of the CA1 pyramidal cells in *Fmr1* KO mice						
	**cell 1**	**cell 2**	**cell 3**	**cell 4**	**cell 5**	**cell 6**
Resting membrane potential (mV)	-67.1	-61	-56	-57.1	-50	-59.3
Input resistance (MΩ)	248.5	210.2	188.4	136.4	162.1	147.7
Sag (%)	18.53					14.5
Time constant (ms)	22.25	29.91	16.47	17.06	20.48	33.52
Rheobase (pA)						
Voltage threshold (mV)						
Depolarization voltage (mV)						
Amplitude action potential (mV)	112.23	98.35	100.54	108.43	83.65	109.37
Action potential duration (ms)						
Single action potential	1.11	1.35	1.19	0.99	0.99	1.25
Firing train (8-12 AP during 500 ms)						
First						
Middle						
Last						
Firing frequency (AP x s-1)						
100 pA						
200 pA						
300 pA						
400 pA						
F/I gain						
ADP/AHP						
ADP single action potential						
Amplitude	5.07	5.55				6.5
Duration	75.25	86.25				100.2
AHP low frequency (<2 Ap x s-1)						
fAHP Amplitude (mV)						
fAHP Duration (ms)						
mAHP Amplitude (mV)						
mAHP Duration (ms)						
AHP firing train (8-12 AP during 500 ms)						
fAHP Amplitude (mV)						
fAHP Duration (ms)						
mAHP Amplitude (mV)						
mAHP Duration (ms)						
mAHP after a firing train (8-12 AP during 500 ms)						
Amplitude (mV)						
Duration (ms)						
Adaptation index						

**Membrane properties of the CA1 pyramidal cells in *Fmr1* KO mice**					
	**cell 7**	**cell 8**	**cell 9**	**cell 10**	**cell 11**
Resting membrane potential (mV)	-63.32	-59.5	-64.3	-71.9	-65
Input resistance (MΩ)	178	231.4	183	141.3	113.2
Sag (%)	12.1	9.93	16.98	19.56	15.11
Time constant (ms)	16.57	16.27	22	14.92	16.65
Rheobase (pA)	70			70	90
Voltage threshold (mV)	-46.44			-52.12	-48.74
Depolarization voltage (mV)	18.07			18.07	16.02
Amplitude action potential (mV)	107.47	83.29	102.64		122.2
Action potential duration (ms)					
Single action potential	1.2	1.09	0.94		0.92
Firing train (8-12 AP during 500 ms)					
First					0.99
Middle					0.97
Last					1
Firing frequency (AP x s-1)					
100 pA	20			16	8
200 pA	32			24	24
300 pA	40			40	32
400 pA	40			44	36
F/I gain	0.11			0.15	0.14
ADP/AHP					
ADP single action potential					
Amplitude	9.43	6.23	5.28		10.31
Duration	82.5	66.85	88.2		68
AHP low frequency (<2 Ap x s-1)					
fAHP Amplitude (mV)	1.46			5.68	6.84
fAHP Duration (ms)	3.1			2.85	4.8
mAHP Amplitude (mV)				4.37	2.76
mAHP Duration (ms)				178.35	222.2
AHP firing train (8-12 AP during 500 ms)					
fAHP Amplitude (mV)					7.23
fAHP Duration (ms)					2.9
mAHP Amplitude (mV)					3.69
mAHP Duration (ms)					69.9
mAHP after a firing train (8-12 AP during 500 ms)					
Amplitude (mV)					0.79
Duration (ms)					69.65
Adaptation index					0.8

**Membrane properties of the CA1 pyramidal cells in *Fmr1* KO mice**					
	**cell 12**	**cell 13**	**cell 14**	**cell 15**	**cell 16**
Resting membrane potential (mV)	-64.8	-69.8	-69.3	-62.5	-60.5
Input resistance (MΩ)	163.4	117.9	148.6	233.1	294.2
Sag (%)	15.38	17.66	12.99	14.76	16.6
Time constant (ms)	22.48	34.34	27.21	22.15	16.88
Rheobase (pA)					
Voltage threshold (mV)					
Depolarization voltage (mV)					
Amplitude action potential (mV)	113.88	120.15	117.6	84.02	91.56
Action potential duration (ms)					
Single action potential	0.95	0.91	0.98	1.27	0.9
Firing train (8-12 AP during 500 ms)					
First	0.99		0.98		
Middle	1.02		1.02		
Last	1.01		1.03		
Firing frequency (AP x s-1)					
100 pA	20	20	12		
200 pA	36	32	24		
300 pA	44	40	32		
400 pA	44	44	40		
F/I gain	0.14	0.14	0.12		
ADP/AHP					
ADP single action potential					
Amplitude	9.74	5.77	4.85	3.85	6.41
Duration	77.75	80.6	92.2	68.15	96.9
AHP low frequency (<2 Ap x s-1)					
fAHP Amplitude (mV)					
fAHP Duration (ms)					
mAHP Amplitude (mV)					
mAHP Duration (ms)					
AHP firing train (8-12 AP during 500 ms)					
fAHP Amplitude (mV)	6.67		9.67		
fAHP Duration (ms)	3.35		2.95		
mAHP Amplitude (mV)	4.18		10.04		
mAHP Duration (ms)	56.5		57.64		
mAHP after a firing train (8-12 AP during 500 ms)					
Amplitude (mV)					
Duration (ms)					
Adaptation index	0.31		0.44		

**Membrane properties of the CA1 pyramidal cells in *Fmr1* KO mice**					
	**cell 17**	**cell 18**	**cell 19**	**cell 20**	**cell 21**
Resting membrane potential (mV)	-60	-66	-68.7	-63	-64.3
Input resistance (MΩ)	177.7	150	143.8	182.9	128.6
Sag (%)	13.02	8.72	11.18	15.98	14.79
Time constant (ms)	25.54	27.3	25.62	24.05	17.67
Rheobase (pA)	10	90	82	70	80
Voltage threshold (mV)	-54	-49.84	-47.08	-45.44	-53.35
Depolarization voltage (mV)	6	18.52	20.82	18.16	13.21
Amplitude action potential (mV)		107.94	109.59	93.45	113.92
Action potential duration (ms)					
Single action potential		0.96	1.03	1.17	0.91
Firing train (8-12 AP during 500 ms)					
First		1	1.1		
Middle		1.03	1.12		
Last		1.03	1.12		
Firing frequency (AP x s-1)					
100 pA	24	8	28	12	8
200 pA	32	36	32	36	32
300 pA	44	48	44	44	48
400 pA	48	52	48	44	56
F/I gain	0.14	0.19	0.12	0.17	0.2
ADP/AHP					
ADP single action potential					
Amplitude		5.71	14.22	10.8	8.03
Duration		120.2	136.9	68.7	85.75
AHP low frequency (<2 Ap x s-1)					
fAHP Amplitude (mV)	9.99	12.21	4.61	8.98	7.08
fAHP Duration (ms)	3.95	4.8	3.77	2.45	3.6
mAHP Amplitude (mV)	7.75	7465	1.4	7.32	2.93
mAHP Duration (ms)	161.65	133.95		131.6	44.4
AHP firing train (8-12 AP during 500 ms)					
fAHP Amplitude (mV)		9.8	4.64		
fAHP Duration (ms)		3.18	3.3		
mAHP Amplitude (mV)		9.43	1.86		
mAHP Duration (ms)		33.8	33.5		
mAHP after a firing train (8-12 AP during 500 ms)					
Amplitude (mV)					
Duration (ms)					
Adaptation index		0.21	0.14		

**Membrane properties of the CA1 pyramidal cells in *Fmr1* KO mice**					
	**cell 22**	**cell 23**	**cell 24**	**cell 25**	**cell 26**
Resting membrane potential (mV)	-63	-69	-65.5	-63.2	-57.2
Input resistance (MΩ)	212.1	156.2	202.9	164	204
Sag (%)	14.11	8.22	8.17		
Time constant (ms)	25.44	26.58	25.87	27.69	22.66
Rheobase (pA)		82		42	12
Voltage threshold (mV)		-49.44		-55.5	-51.2
Depolarization voltage (mV)		17.21		9.49	6.5
Amplitude action potential (mV)	87.53	101.9	86.85	119.11	105.29
Action potential duration (ms)					
Single action potential	1.24	1.07	1.19	1.2	0.93
Firing train (8-12 AP during 500 ms)					
First			1.15		0.99
Middle			1.16		1
Last			1.15		1
Firing frequency (AP x s-1)					
100 pA		8	28		16
200 pA		36	44		52
300 pA		44	52		60
400 pA		52	56		60
F/I gain		0.18	0.15		0.19
ADP/AHP					
ADP single action potential					
Amplitude	8.94	2.44	8.7	6.62	4.49
Duration	83.7	51	131.75	80.8	72.85
AHP low frequency (<2 Ap x s-1)					
fAHP Amplitude (mV)		4.93		5.1	7
fAHP Duration (ms)		4.25		2.9	3.8
mAHP Amplitude (mV)		1.74		2.78	2.75
mAHP Duration (ms)		179.2		39.35	286.45
AHP firing train (8-12 AP during 500 ms)					
fAHP Amplitude (mV)			4.41		7.21
fAHP Duration (ms)			3.3		3.55
mAHP Amplitude (mV)			2.35		3.69
mAHP Duration (ms)			40.95		46.9
mAHP after a firing train (8-12 AP during 500 ms)					
Amplitude (mV)					
Duration (ms)					
Adaptation index			0.46		0.48

**Membrane properties of the CA1 pyramidal cells in *Fmr1* KO mice**					
	**cell 27**	**cell 28**	**cell 29**	**cell 30**	**cell 31**
Resting membrane potential (mV)	-58.7	-60	-70	-59.3	-69
Input resistance (MΩ)	260	125	97	200	130
Sag (%)					
Time constant (ms)	24.38	26.24	19.91	21.85	25.47
Rheobase (pA)	50	62	128	10	150
Voltage threshold (mV)	-42.9	-42.48	-51.4	-49.7	-47.27
Depolarization voltage (mV)	18.28	19.26	17.49	8.5	21.79
Amplitude action potential (mV)	109.68	125.76	123.77	118.23	
Action potential duration (ms)					
Single action potential	1.25	0.98	0.96	1.04	
Firing train (8-12 AP during 500 ms)					
First		0.98	1	0.96	
Middle		0.99	1.02	0.99	
Last		1	1.03	1	
Firing frequency (AP x s-1)					
100 pA	20	8	8	20	
200 pA	40	16	12	36	
300 pA	44	28	24	40	
400 pA	44	36	32	44	
F/I gain	0.19	0.1	0.12	0.12	
ADP/AHP					
ADP single action potential					
Amplitude	7.11	11.51	5.28	2.11	
Duration	79.4	82.15	95.85	254.45	
AHP low frequency (<2 Ap x s-1)					
fAHP Amplitude (mV)	5.71	12.84	12.11	12.91	5.58
fAHP Duration (ms)	1.96	3.08	3.9	3.7	2.65
mAHP Amplitude (mV)	4.09	3.39	8.64	3.88	6.07
mAHP Duration (ms)	201.4	100.85	142.3	205.25	128.95
AHP firing train (8-12 AP during 500 ms)					
fAHP Amplitude (mV)		9.53	8.36	9.9	
fAHP Duration (ms)		3.8	3.45	2.6	
mAHP Amplitude (mV)		8.29	7.83	8.9	
mAHP Duration (ms)		44.8	48.3	55.15	
mAHP after a firing train (8-12 AP during 500 ms)					
Amplitude (mV)		1.95	1.3		
Duration (ms)		185.45	147.3		
Adaptation index		0.42	0.35		

Membrane properties of the CA1 pyramidal cells in *Fmr1* KO mice	
	**cell 32**
Resting membrane potential (mV)	-58.4
Input resistance (MΩ)	140
Sag (%)	
Time constant (ms)	33.18
Rheobase (pA)	40
Voltage threshold (mV)	-46.6
Depolarization voltage (mV)	10.22
Amplitude action potential (mV)	113.37
Action potential duration (ms)	
Single action potential	1
Firing train (8-12 AP during 500 ms)	
First	1.09
Middle	1.12
Last	1.11
Firing frequency (AP x s-1)	
100 pA	12
200 pA	20
300 pA	32
400 pA	36
F/I gain	0.14
ADP/AHP	
ADP single action potential	
Amplitude	5.43
Duration	88.7
AHP low frequency (<2 Ap x s-1)	
fAHP Amplitude (mV)	7.57
fAHP Duration (ms)	4.35
mAHP Amplitude (mV)	3.14
mAHP Duration (ms)	146.9
AHP firing train (8-12 AP during 500 ms)	
fAHP Amplitude (mV)	7.51
fAHP Duration (ms)	2.52
mAHP Amplitude (mV)	7.2
mAHP Duration (ms)	66.85
mAHP after a firing train (8-12 AP during 500 ms)	
Amplitude (mV)	1.53
Duration (ms)	164.75
Adaptation index	0.73
